# Real Lives and White Lies in the Funding of Scientific Research: The granting system turns young scientists into bureaucrats and then betrays them

**DOI:** 10.1371/journal.pbio.1000197

**Published:** 2009-09-15

**Authors:** Peter A. Lawrence

**Affiliations:** Department of Zoology, University of Cambridge and Medical Research Council Laboratory of Molecular Biology, Cambridge, United Kingdom

*“O, how full of briers is this working-day world!”—William Shakespeare, “As You Like It”*

It is a summer day in 2009 in Cambridge, England, and K. (39) looks out of his lab window, wondering why he chose the life of a scientist [1]. Yet it had all begun so well! His undergraduate studies in Prague had excited him about biomedical research, and he went on to a PhD at an international laboratory in Heidelberg. There, he had every advantage, technical and intellectual, and his work had gone swimmingly. He had moved to a Wellcome-funded research institute in England in 1999. And although his postdoc grant, as is typical, was for only two years, he won a rare career development award that gave him some independence for four more years. A six-year postdoc was an unusual opportunity, and it allowed him to define his own research field. By 2004, he had published six experimental papers in good journals, and on four of these, he was first author. It was the high point in his career, and when he applied for posts in Cambridge, London, Stanford, and Tubingen, he was short-listed for them all. He chose Cambridge University and a Royal Society Research Fellowship that offered him up to ten years' salary. This should have brought the peace of mind to plan projects that would take five years, or even longer.

So, what went wrong? It had taken almost a year to prepare, submit, and be awarded his initial research grant (for late 2005–late 2008) from a publicly funded agency in the UK, the Biotechnology and Biological Sciences Research Council (BBSRC). Immediately, he hired a technician and started to train her carefully. During early 2006, he took on a postdoc, Frieda, and a student, and they both settled in well. However, by mid 2007, K. began to worry about his future: although the BBSRC grant had run for less than two years, it was already high time to apply for another. He submitted a new application in October 2007 and, although it was well reviewed and received a high rating, he found out in spring 2008 that it was not funded. As an insurance, he had concocted a different project (you cannot submit to different agencies with the same plans) and sent it to Cancer Research, UK, in February 2008—this application was also excellently reviewed but also turned down in August 2008. Now he was near the end of his initial grant, and his technician, who had a family to support, left to take a more secure job in a nearby research institute—laying waste to all her specialised knowledge that they had both worked so hard to build. Soon, Frieda's postdoc grant was about to end, so K. applied to several local colleges and trusts to keep her going, but won her only another 6 months' salary.

Becoming anxious and not sleeping well, he had approached the Wellcome Trust in the autumn of 2008 with a rewritten and updated version of the rejected BBSRC project. But, with her extra 6 months over in April 2009 and no security (she has two small children), Frieda had been concentrating less and less on her work; she reluctantly abandoned her lifelong ambition to be a researcher. She looked for a job in science publishing or in the granting agencies—both of which, ironically, offer better working conditions and much better security of employment than research. That morning, Frieda had been offered a post as assistant editor of a journal, and it was time to organise her leaving party. So, on this summer day in 2009, K. has one student left in his group and has one grant application outstanding. If he gets the grant, the salary dedicated for Frieda could be given to a new postdoc, but that person would have to be trained all over again. Yes, the second half of 2007 and all of 2008 had been a nightmare—14 of these 18 months had been almost entirely devoted to writing grant applications. K. now sees how he has changed from being an enthusiastic scientist into an insecure bureaucrat. He feels he has lost much of his last 3 years and wasted his BBSRC grant, despite doing his very best (see Box 1).

**Box 1. Scientists Speak Out: A Broken System**

“What a strange business this is: We stay in school forever. We have to battle the system with only a one in eight or one in ten chance of getting funded. We give up making a living until our forties. And we do it because we want to help the world. What kind of crazy person would go for that?”*—Nancy Andrews, Vice Chancellor for Academic Affairs and Dean of the Duke University School of Medicine*

“The present fashion is for BIG projects, generated from above. This view was boosted by the Human Genome Project, and while some big projects work, they erode the anarchic and creative spirit that drives science forward. Students and post-docs see this, and it is hard for their idealism to persist.”*—Marvin Wickens, Max Perutz Professor of Molecular Biology and Biochemistry, University of Wisconsin-Madison*

“The problem is, over and over again, that many very creative young people, who have demonstrated their creativity, can't figure out what the system wants of them—which hoops should they jump through? By the time many young people figure out the system, they are so much a part of it, so obsessed with keeping their grants, that their imagination and instincts have been so muted (or corrupted) that their best work is already behind them. This is made much worse by the US system in which assistant professors in medical schools will soon have to raise their own salaries. Who would dare to pursue risky ideas under these circumstances? Who could dare change their research field, ever?”*—Ted Cox, Edwin Grant Conklin Professor of Biology, Director of the Program on Biophysics, Princeton University*

“One cannot run a small one-grant lab here—there just aren't enough funds in a single NIH grant (which typically provides about $225,000 in direct costs/year) to pay for a PI salary (especially at medical centers), plus supplies, plus the salaries of one or two other people (not to mention tuition, which we also have to pay some part of for grad students). Even with three grants—which are mighty hard to get and harder to maintain—so much money goes for salaries that there is barely enough left over to do an experiment. So the system demands writing more and more grants and doing fewer and fewer experiments. I feel like I'm always writing some summary, justifying how some small pot of money was spent, and how this endeavor differs from the other ongoing efforts in my lab (most agencies of course want to believe that they are the sole source of funding for a particular project). In other words, the system just doesn't work”*—Richard Mann, Professor, Department of Biochemistry and Molecular Biophysics, Columbia University*

“After 30 or so years of research, I went through a period of two and a half years without funding, despite eight applications. Last year, I did finally achieve a further three years funding with two grant applications. But what was achieved by this delay? Programs that should have begun over 2 years ago have been held back, allowing our competitors in other countries to get ahead. The funded grants are no better than those that were turned down.”*—Michael Glazer, Professor of Physics, University of Oxford*

“You can write a grant for an important project for which there is ample pilot data. It can be very well reviewed but still fail to be funded. Money is limited, and maybe the projects funded are even better, so you cannot necessarily complain. But the issue is wastage. The pilot data may go nowhere—just languish in a drawer. Expertise will be lost—the applicants will have to work on something else. Eventually, someone else may repeat the work and bring it to publication, but without the advantage of knowing what had already been done. So the previous investment of the funding bodies is wasted.”*—David Strutt, Senior Research Fellow, Professor, Department of Biomedical Science, University of Sheffield*

“People do not realize that when it comes to arguing their case for more funding, scientists who do basic research are the least articulate, least organized, and least temperamentally equipped to justify what they are doing. In a society where selling is so important, where the medium is the message, these handicaps can spell extinction.”*—Arthur Kornberg, Nobel Laureate in Physiology or Medicine (deceased 2007)*

Editor's note: The above comments were e-mailed to the author, with the exceptions of the quotes from Nancy Andrews (see [4]) and Arthur Kornberg (see [15]).

K.'s plight (an authentic one) illustrates how the present funding system in science eats its own seed corn [2]. To expect a young scientist to recruit and train students and postdocs as well as producing and publishing new and original work within two years (in order to fuel the next grant application) is preposterous. It is neither right nor sensible to ask scientists to become astrologists and predict precisely the path their research will follow—and then to judge them on how persuasively they can put over this fiction. It takes far too long to write a grant because the requirements are so complex and demanding. Applications have become so detailed and so technical that trying to select the best proposals has become a dark art. For postdoctoral fellowships, there are so many arcane and restrictive rules that applicants frequently find themselves to be of the wrong nationality, in the wrong lab, too young, or too old. Young scientists who make the career mistake of concentrating on their research may easily miss the deadline for the only grant they might have won. Research institutes with their own funds can solve these problems, but grant holders like K. do not have any flexibility. The real world of science has no tidy banks of pigeonholes, each one occupied for a standard period by an exemplary student or a perfect postdoc.

## Grantsmanship and the Application Process

*“Scientists might have had a Hippocratic oath of their own. They might have promised their gifts to mankind. But instead, I have fathered a race of inventive dwarfs who can be hired for anything.”—Bertolt Brecht “The Life of Galileo,” version by David Hare*

After more than 40 years of full-time research in developmental biology and genetics, I wrote my first grant and showed it to those experienced in grantsmanship. They advised me my application would not succeed. I had explained that we didn't know what experiments might deliver, and had acknowledged the technical problems that beset research and the possibility that competitors might solve problems before we did. My advisors said these admissions made the project look precarious and would sink the application. I was counselled to produce a detailed, but straightforward, program that seemed realistic—no matter if it were science fiction. I had not mentioned any direct application of our work: we were told a plausible application should be found or created. I was also advised not to put our very best ideas into the application as it would be seen by competitors—it would be safer to keep those ideas secret.

The peculiar demands of our granting system have favoured an upper class of skilled scientists who know how to raise money for a big group [3]. They have mastered a glass bead game that rewards not only quality and honesty, but also salesmanship and networking. A large group is the secret because applications are currently judged in a way that makes it almost immaterial how many of that group fail, so long as two or three do well. Data from these successful underlings can be cleverly packaged to produce a flow of papers—essential to generate an overlapping portfolio of grants to avoid gaps in funding.

Thus, large groups can appear effective even when they are neither efficient nor innovative. Also, large groups breed a surplus of PhD students and postdocs that flood the market; many boost the careers of their supervisors while their own plans to continue in research are doomed from the outset. The system also helps larger groups outcompete smaller groups, like those headed by younger scientists such as K. It is no wonder that the average age of grant recipients continues to rise [4]. Even worse, sustained success is most likely when risky and original topics are avoided and projects tailored to fit prevailing fashions—a fact that sticks a knife into the back of true research [5]. As Sydney Brenner has said, “Innovation comes only from an assault on the unknown” [6].

How did all this come about? Perhaps because the selection process is influenced by two sets of people who see things differently. The first are the granting organisations whose employees are charged to spend the money wisely and who believe that the more detailed and complex the applications are, the more accurately they will be judged and compared. Over the years, the application forms have become encrusted with extra requirements.

Universities have whole departments devoted to filling in the financial sections of these forms. Liaison between the scientists and these departments and between the scientists and employees of the granting agencies has become more and more Kafkaesque.

The second set of people are the reviewers and the committee, usually busy scientists who themselves spend much time writing grants. They try to do their best as fast as they can. Generally, each reviewer reads just one or two applications and is asked to give each a semiquantitative rating (“outstanding,” “nationally competitive,” etc.). Any such rating must be whimsical because each reviewer sees few grants. It is particularly difficult to rank strongly original grants; for no one will know their chances of success. The committee are usually presented with only the applications that have received uniformly positive reviews—perhaps favouring conventional applications that upset no one. The committee might have 30 grants to place in order of priority, which is vital, as only the top few can be funded. I wonder if the semiquantitative and rather spurious ratings help make this ordering just [7]. I also suspect any gain in accuracy of assessment due to the detail provided in the applications does not justify the time it takes scientists to produce that detail.

## How to “Restore Science to Its Rightful Place” [8]

*“Could we stimulate more discovery and creativity if more scientists had…security of…research support? Would this encourage risk-taking and lead to an overall improvement in the quality of science?”—Stephen Quake (Stanford University)* [*9*] *.*

K.'s problems were partly due to the time it took to write his applications and the long lag period between submission and decision. Drastic simplification of this grant-writing process would help scientists return to the business of doing science. Grant applications should be much shorter, and if so, scientists would spend less time writing them and less time reviewing other applications. Since the failure rate is high, these savings of time and effort would be very substantial—in Cambridge University, an average scientist writes about three applications to get one grant; similarly, in the United States “after multiple submissions and a protracted process, only about 20% of grants will ultimately be funded” [4], and the success rate is dropping [10] (see Box 2).

**Box 2. Scientists Speak Out: A Path to Reform**

“I long ago concluded that grant applications should be short, they should contain ideas, they should have a short turn-around time, and they should invest in a person who has shown they can do original and interesting work. Shortness has one very important additional virtue not often noticed: a reviewer can actually remember what they've read. A typical application in this country runs to 25 pages, they are laden with detail, and they select in the long run for referees who can remember detail, a terrible outcome.”*—Ted Cox, Princeton University*

(Author's note: the National Institutes of Health has recently reduced the page limit on applications from 25 to 12–still far too long, in my opinion.)

“My solution? Everyone should get slotted into a funding category and assessed every five years. If you're productive, you get five more years of resources. If productivity is down, you are moved down a category. If it is high, you can apply to move up. Starting PIs are in a different category and must apply to get onto the treadmill. The difference: PIs would be judged by overall productivity, not grantsmanship. We can stop wasting our time writing grants, and the system can be more easily calibrated to train a sustainable number of postdocs. It is depressing to train people who will struggle for funding.

A peer-reviewed, 5-year renewable, productivity-based ‘track’ system with a set amount of money at each level would stabilize funding, encourage innovation and productivity, allow each PI to control how their money is allocated, and permit us to make nationwide decisions about the size of our science enterprise. It also has the merit of simplicity.”*—Ross Cagan, Professor of Developmental and Regenerative Biology, Mount Sinai School of Medicine*

“A simpler, more efficient, fairer, and more productive system is that operated by research institutes, such as the IMCB in Singapore, where investigators are given a budget, allowed to get on with their research and reviewed after five years.”*—Philip Ingham, Professor, Institute of Molecular and Cell Biology, Singapore/MRC Centre for Developmental and Biomedical Genetics, University of Sheffield*

“I commend the European Research Council: the grants are larger and last longer. They are judged purely on scientific merit by scientists, no political strings, no fake collaborations, no age limits.”*—Maria Leptin, Professor at the Institute of Genetics, University of Cologne, Germany, Director elect, European Molecular Biology Organization*

“It would be far more honest if we reverted to the system of direct funding to the universities. I would like to see future scientists taken off this treadmill so that they could be scientists, not fund raisers. In the UK, it was under the Thatcher government that the move was made away from direct funding towards indirect funding via the research councils. It was this move, coupled with the ideology of ‘wealth creation,’ that created the problems we have today.

But how to persuade government and the public that scientists should simply be given money for research? We would need an intense program of reeducation about how science works in practice; emphasis would have to shift from plans to outcomes. Thus, most science would be funded through local channels (the university, the department, etc.) and, every four to five years, the retrospective judgment of our results would drive future funding. This would be true peer review rather than the present ‘prejudicial review.’ After all, we do expect to be peer reviewed when we submit a manuscript—for work that we have already done. No one writes a paper about what they intend to do. But there would be resistance to these changes, partly because many colleagues are experts at playing the present (dishonest) system.”*—Michael Glazer, University of Oxford*

Editor's note: The above comments were e-mailed to the author.

It would help free up time for research if scientists were not forced to dream up future plans but instead could opt to present their recent publications. Even some first-time applicants with a strong recent record, like K., could take this option; others might prefer to write projects. Grants should last for longer; three years is too short, and having five-year renewals would itself reduce the burden of grant writing.

There should be a presumption against large groups, and people who aim to run them should demonstrate both efficiency and effectivity; they should show that they have enough time to run each of their grants and to care for each of their people. We need to reduce the bureaucracy as well as the delays it generates: the right approach to too much paperwork is not to appoint expensive offices to cope [11], but to cull it. Rationalisation would save a lot of money that could be put into the grants themselves rather than into their administration.

An important reform would be to set a maximum limit on the number of papers that could be offered as part of any application or any assessment. At the moment, evaluating individuals and departments rewards those who produce many articles, mainly because counting papers is so much simpler than reading them. Over 20 years, this mismeasurement of science [12] has wrought a sea change in practice: no longer are communication and record the primary purposes of publishing; instead, we now use papers as tokens to get jobs and funding. This same sea change has fuelled a huge increase in the number of papers and journals and decimated their quality and utility. The solution is to allow, say, only three to five of the best papers of the group from over the previous five years to be offered for assessment (as Howard Hughes Medical Institute already do in the US). The evaluation of these papers should be corrected for the size of the group, i.e., productivity would be rated per person, not per group. If these reforms were enacted, the pressure to rush out many papers would be replaced by pressure to complete projects that report stories of value and present them well. In consequence, the literature would be transformed and improved, and we would all benefit. But this reform would create a problem. At the moment, young people need a paper as a ticket for the next step, and we should therefore give deserving, but unlucky, students another chance. One way would be to put more emphasis on open interviews (with presentation by the candidate and questions from the audience) and references. Not objective? No, but only false objectivity is offered by evaluating real people using unreal calculations with numbers of papers, citations, and journal impact factors. These calculations have not only demoralised and demotivated the scientific community [13], they have also redirected our research and vitiated its purpose [14].
